# Comprehensive genotyping of the USA national maize inbred seed bank

**DOI:** 10.1186/gb-2013-14-6-r55

**Published:** 2013-06-11

**Authors:** Maria C Romay, Mark J Millard, Jeffrey C Glaubitz, Jason A Peiffer, Kelly L Swarts, Terry M Casstevens, Robert J Elshire, Charlotte B Acharya, Sharon E Mitchell, Sherry A Flint-Garcia, Michael D McMullen, James B Holland, Edward S Buckler, Candice A Gardner

**Affiliations:** 1 Institute for Genomic Diversity, Biotechnology bldg., Cornell University, Ithaca, NY, 14853, USA; 2 USA Department of Agriculture (USDA) - Agricultural Research Service (USDA-ARS; 3 North Central Regional Plant Introduction Station, Agronomy bldg., Department of Agronomy, Iowa State University, Ames, IA, 50001, USA; 4 Bioinformatics Research Center, Thomas Hall, North Carolina State University, Raleigh, NC, 27606, USA; 5 Department of Plant Breeding and Genetics, Bradfield Hall, Cornell University, Ithaca, NY, 14853, USA; 6 Division of Plant Sciences, Curtis Hall, University of Missouri, Columbia, MO, 65211,USA; 7 Department of Crop Science, Williams Hall, North Carolina State University, Raleigh, NC, 27695, USA

**Keywords:** Diversity, Genotyping by sequencing, Germplasm, Maize, Public

## Abstract

**Background:**

Genotyping by sequencing, a new low-cost, high-throughput sequencing technology was used to genotype 2,815 maize inbred accessions, preserved mostly at the National Plant Germplasm System in the USA. The collection includes inbred lines from breeding programs all over the world.

**Results:**

The method produced 681,257 single-nucleotide polymorphism (SNP) markers distributed across the entire genome, with the ability to detect rare alleles at high confidence levels. More than half of the SNPs in the collection are rare. Although most rare alleles have been incorporated into public temperate breeding programs, only a modest amount of the available diversity is present in the commercial germplasm. Analysis of genetic distances shows population stratification, including a small number of large clusters centered on key lines. Nevertheless, an average fixation index of 0.06 indicates moderate differentiation between the three major maize subpopulations. Linkage disequilibrium (LD) decays very rapidly, but the extent of LD is highly dependent on the particular group of germplasm and region of the genome. The utility of these data for performing genome-wide association studies was tested with two simply inherited traits and one complex trait. We identified trait associations at SNPs very close to known candidate genes for kernel color, sweet corn, and flowering time; however, results suggest that more SNPs are needed to better explore the genetic architecture of complex traits.

**Conclusions:**

The genotypic information described here allows this publicly available panel to be exploited by researchers facing the challenges of sustainable agriculture through better knowledge of the nature of genetic diversity.

## Background

Maize (*Zea mays *L.) is one of the most important crops in the world, being one of the main sources of human food, animal feed, and raw material for some industrial processes [1].Furthermore, maize is a significant model plant for the scientific community to study phenomena such as hybrid vigor, genome evolution, and many other important biological processes. The maize genome is complex, and has a very high level of genetic diversity compared with other crops and model plant species [2]. The *Zea *genome is in constant flux, with transposable elements changing the genome and affecting genetic diversity [3]. Structural variations between any two maize plants are prevalent and are enriched relative to single-nucleotide polymorphism (SNP) markers as significant loci associated with important phenotypic traits [4]. The availability of new sequencing technologies at increasingly affordable prices has provided the opportunity to investigate more deeply the maize genome and its diversity, enabling genome-wide association studies (GWAS) and genomic selection (GS) strategies.

Since the beginning of the 20th Century, when Shull [5] and East [6] first investigated inbreeding and heterosis in maize, breeding programs around the world have developed maize inbred lines using diverse strategies. The USDA-ARS North Central Regional Plant Introduction Station (NCRPIS) in Ames, Iowa, an element of the National Plant Germplasm System, along with germplasm banks around the world, has conserved distinct inbred lines that represent nearly a century of maize breeding efforts. Researchers have genotypically characterized subsets of these maize inbred lines to assist with curatorial management of germplasm collections, to evaluate diversity within breeding programs, and for use in association mapping [7-10]. Some association panels have been used successfully to characterize many different traits, frequently through a candidate gene strategy [11]. However, the sample sizes used in these studies may not have been large enough to detect all of the key quantitative trait loci (QTL) for the complex traits. Furthermore, the nature of population structure in maize may have resulted in further dilution of statistical power and high rates of false discovery [12]. In addition, candidate gene strategies require an understanding of the biochemical or regulatory pathways controlling the traits.

Recently, Elshire *et al. *[13] developed a simple new sequencing procedure that provides a large number of markers across the genome at low cost per sample. The approach, called genotyping by sequencing (GBS), can be applied to species with high diversity and large genomes such as maize. It does not rely on previous knowledge of SNPs; however, the high-quality reference genome for the maize inbred *B73 *[14] is used at this point to anchor the position of the SNPs. The method enables characterization of germplasm collections on a genome-wide scale, and greatly expands the number of individuals and markers under study, which then increases the chances of discovering more uncommon or rare variants [15]. In maize, there are examples of important rare alleles unique to some groups of germplasm, such as alleles at *crtRB1 *that increase β-carotene concentrations in kernels [16]. Several studies have also suggested that rare alleles could explain the 'missing heritability' problem. This is the phenomenon by which a large portion of the inferred genetic variance for a trait is often not fully accounted for by the loci detected by GWAS [17]. Moreover, the increased number of samples and markers allow a deeper study of haplotype structures and linkage disequilibrium (LD). Regions with strong LD and large haplotype blocks as a result of reduced recombination make it more difficult to separate genes that can have different effects, affecting both mapping and/or selection of the positive alleles for a trait. This linkage between favorable and negative alleles also contributes to heterosis [18].

In the current study, we used GBS to analyze a total of 4,351 maize samples from 2,815 maize accessions with 681,257 SNP markers distributed across the entire genome. These data allowed us to 1) compare this new sequencing technology with other available options, 2) explore the potential of this new technology to help with curation and use of germplasm, 3) evaluate genetic diversity and population structure both across the genome and between groups of germplasm, 4) investigate the history of recombination and LD through the different breeding groups, and 5) explore the potential of the collection as a resource to study the genetic architecture of quantitative traits.

## Results

### Marker coverage and missing data

**Figure 1:**
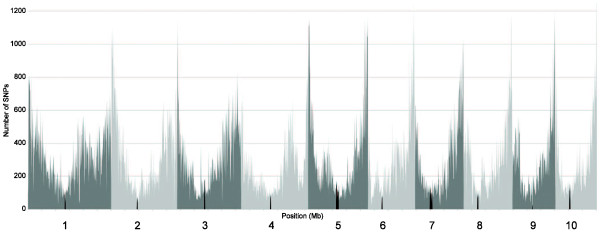
**distribution of single-nucleotide polymorphisms (SNPs) across the genome**. Distribution of the number of SNPs found in 1 Mb windows across the 10 maize chromosomes. Centromere positions are shown in black.

The germplasm set examined in this experiment comprised 2,711 available maize inbred accessions preserved in the USDA-ARS NCRPIS collection (some of them with more than one source), another 417 candidates to be incorporated into the USDA collection as new sources of diversity, and the 281 maize inbred lines from the Goodman maize association panel [8]. Most of the accessions were sequenced once, with one representative plant chosen for the DNA extraction, resulting in a single GBS sample. However, for 558 accessions, more than one plant was sequenced so different sources could be compared, and therefore more than one GBS sample was available. Moreover, 326 DNA samples were sequenced multiple times as technical replicates. Thus, the total number of GBS samples analyzed in this study was 4,351 (see Additional file 1). From the complete set of 681,257 SNP markers across all maize lines analyzed to date, we selected 620,279 SNPs that are polymorphic among our samples. These SNPs are distributed along the 10 maize chromosomes, and more highly concentrated in sub-telomeric than pericentromeric regions (Figure 1).

The average base-call error rate based on repeated samples was 0.18%. An additional level of quality control was provided by approximately 7,000 SNPs that overlapped with those obtained with a large genotyping array [19] for the 281 maize inbreds from the Goodman association panel. The mean discrepancy rate between the GBS and array SNP genotypes for all calls was 1.8%. When heterozygote calls are excluded from the comparison, the discrepancy rate decreased to 0.58%.

The average coverage (SNP call rate) by sample was 35%, with values ranging from 2 to 75%. However, when samples were sequenced more than once, coverage improved substantially. For example, the Goodman association panel was evaluated twice, and reduced the average missing data from 63% based on a single run to 35% for the merged data. The nested association mapping (NAM) parents [18], covered by seven replicate sequencing runs, was found to have only 23% missing data. The inbred line *SA24*, used as a check, was analyzed more than 25 times and had only 16% missing data. In addition, coverage was highly dependent on the genotype. A substantial number of the total reads could not be aligned to the reference genome, some because of limited sensitivity of the Burrows-Wheeler Alignment (BWA) software, but most because of presence/absence variation (PAV). Use of the *B73 *reference genome resulted in inbreds more closely related to *B73 *achieving values of less than 20% missing data with only two samples, whereas more distant inbreds maintained values of around 30% missing data even after several replicate sequencing runs.

Imputation of missing data was performed using an algorithm that searched for the closest neighbor in small SNP windows across our entire maize database (approximately 22,000 *Zea *samples), allowing for a 5% mismatch. If the requirements were not met, the SNP was not imputed, leaving only about 10% of the data unimputed. When comparing the imputed GBS data with the results from the genotyping array [19] for the 281 maize inbreds from the Goodman association panel, the median discrepancy rate for all calls was 4%. Excluding heterozygote calls, the median error rate was 1.83%. Imputed data were used only to perform GWAS analysis.

### Integrity and pedigree relationships of the germplasm collection

**Figure 2:**
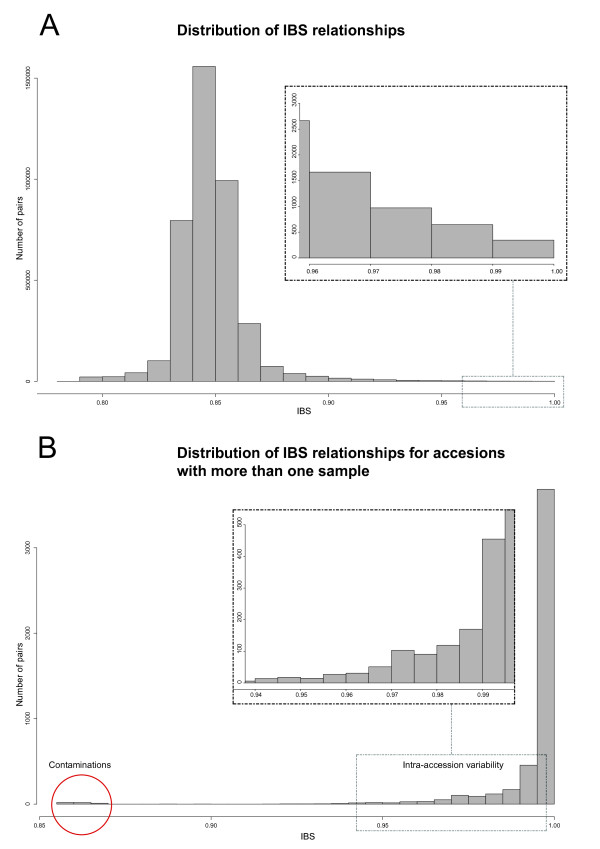
**Identical by state (IBS) distribution across GBS samples**. Distribution of IBS values across **(A) **the 2,815 accessions and **(B) **for accessions with multiple samples.

Curatorial management of such an enormous collection of an annual plant is challenging, and various steps of the process may contribute to problems such as errors or material duplications. However, when we calculated the proportion of markers identical by state (IBS) for all pairs of lines (Figure 2A), GBS data showed that more than 98% of the approximately 2,200 samples that shared an accession name were more than 0.99 IBS even when derived from different inventory samples (Figure 2B). Most of the mismatches were traced back to problems during the DNA manipulation step. This showed that misclassification or contamination problems are not common in the bank. When more than one sample per accession was available, intra-accession variability was detected (Figure 2B). For those accessions, the IBS value was lower than expected, owing to residual heterozygosity. However, for most of the accessions in this study, only one plant was analyzed, and thus intra-accession variability could not be assayed. Based on our average error rates, we selected 0.99 as a conservative value to assume that two different samples with the same name but different origins are actually the same accession. When more than two samples per accession were available, if IBS values were consistent between all comparisons we considered the differences to be the result of residual heterozygosity. We merged the information from replicated samples that met those criteria to obtain a final list of 2,815 unique maize inbred lines.

**Figure 3:**
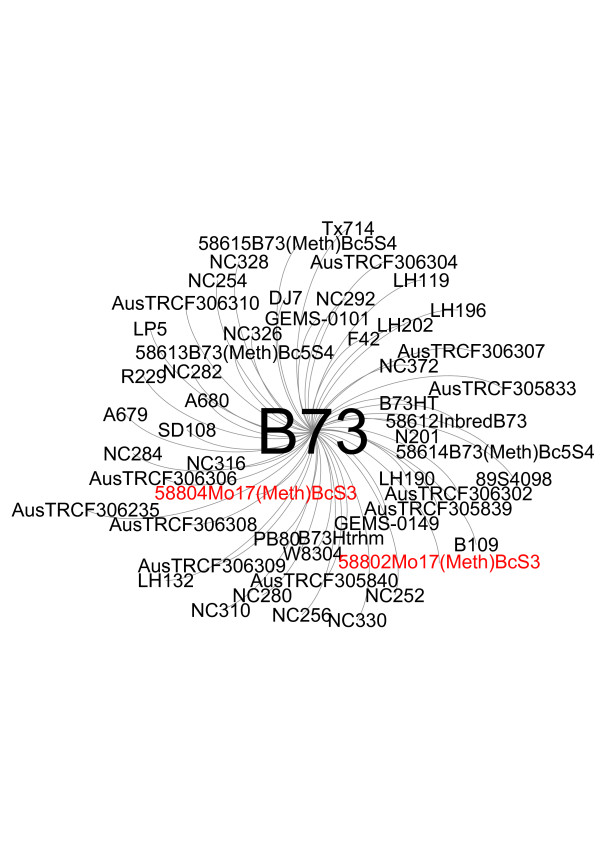
***B73 *network diagram**. Network relationships of maize inbred lines with values of IBS greater than 0.97 for *B73*.

Maize inbred development through the world has been accomplished in many different ways, but some of the most common procedures consist of intermating existing elite materials or incorporating a desirable trait from a donor into an elite inbred line through backcross breeding [20]. Thus, we expected that a high number of the inbred lines in our collection would be closely related. Using IBS, we examined the distribution of the IBS relationships (Figure 2A) and the 10 closest neighbors for each unique inbred line (see Additional file 2). The data reflect the continuous exchange and refinement of germplasm that has occurred over the breeding history of maize and the efforts by breeders to introduce new diversity into their programs. We calculated identity by descent (IBD) for all possible pairwise combinations of the inbreds, and found that 603 lines (21% of the collection) had at least one other accession that was 97% identical (equal to the relationship expected between a parental inbred and a progeny derived by four backcrosses to that parent). For some of the more historically important inbred lines, the number of relationships exceeded 10. For example, *B73 *shares more than 97% of its genome with more than 50 inbreds (Figure 3), congruent with its contribution to the pedigrees of many important commercial lines [21].

The network of relationships obtained using GBS data (see Additional file 3), combined with pedigree information, provides a tool to identify anomalies and potential errors in the identity of accessions. These data, in hands of experts on maize germplasm (for example, the USDA maize curator), can be used to identify accessions that may have been misclassified, select best sources for multiplication/distribution, eliminate duplications, select core collections, add or recommend new experimental entries, and in theory, to assess genetic profile changes over successive regenerations, another quality-assurance measure.

### Population structure

**Figure 4:**
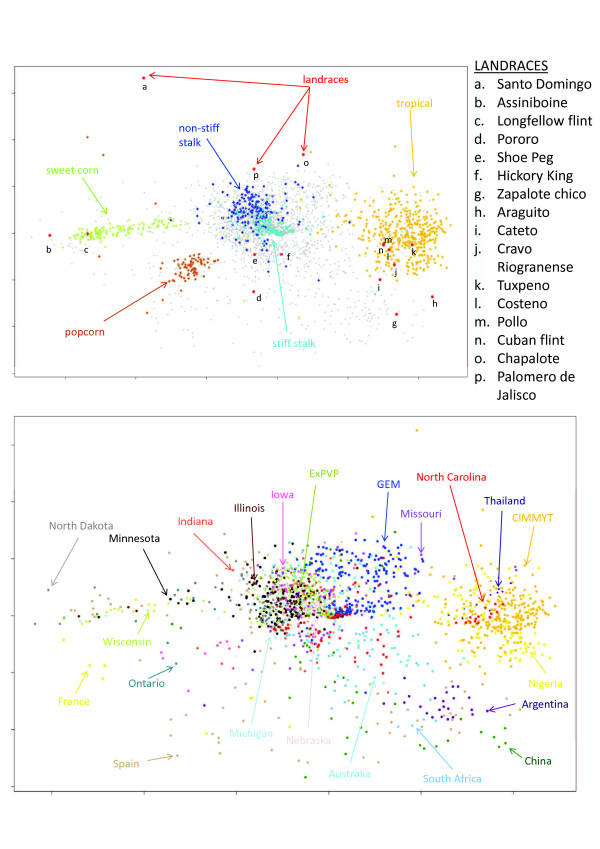
**Multidimensional scanning for 2,815 maize inbred lines**. Genetic relationships between the maize inbred lines preserved at the NCRPIS germplasm bank visualized using a principal coordinate analysis of the distances matrix. The × and Y axes represent PCo1 and PCo2 respectively. Colors are assigned based on **(A) **population structure or **(B) **breeding program. Inbred lines obtained directly from landraces without selection are highlighted in red to serve as reference.

Maize lines from breeding programs with different objectives and environments were included in our final set of lines (see Additional file 1). It is expected that different groups of germplasm will result in population stratification [7,8]. An analysis of the similarity matrix using principal coordinate analysis (PCoA) with a multidimensional scaling (MDS) plot showed that GBS data could describe the genetic variation among our breeding lines in accordance with their known ancestral history (Figure 4A). For example, the inbreds grouped into different subpopulations along the PCo1 axis, with tropical materials on one side, and sweet corn, derived from Northern Flint materials, on the other.

When the inbreds were classified according to breeding program of origin (Figure 4B), the different breeding programs also tended to group together, with most of the USA programs in the two major germplasm groups recognized by temperate maize breeders (referred to as stiff stalk and non-stiff stalk [21]). However, some USA inbred lines (for example, the temperate-adapted all-tropical lines developed at North Carolina State University) were found to be interspersed with tropical lines from CIMMYT (the International Maize and Wheat Improvement Center), while others (for example, the semi-exotic inbreds from the Germplasm Enhancement of Maize (GEM) program, derived from crossing USA and tropical lines) were located between the stiff stalk/non-stiff stalk and the tropical clusters. Finally, other materials from international programs (for example, Spain, France, China, Argentina, or Australia) seem to represent germplasm pools different from those commonly used in North American programs. As expected, these usually did not form clusters with any of the other groups.

### Distribution of alleles and allele frequencies

**Figure 5:**
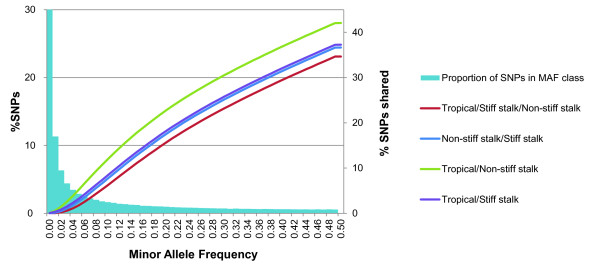
**Minor allele frequency (MAF) distribution and percentage of single-nucleotide polymorphisms (SNPs) shared between maize subpopulations**. Histogram of MAF distribution over all groups, and cumulative percentage of SNPs shared between different groups of germplasm for each class of MAF. Columns represent the percentage of SNPs in each MAF category; lines represent the percentage of alleles shared between the groups of germplasm at equal or lesser MAF value.

The site frequency spectrum (SFS) for the entire collection showed that most of the SNPs in the Ames inbred panel (68%) had a minor allele frequencies (MAF) less than 0.1, with more than half of all SNPs being rare (MAF < 0.05) (Figure 5). This result suggests that some alleles might be unique to different subgroups of germplasm. To compare levels of diversity between different germplasm groups, we analyzed the percentage of alleles present in those groups. The inbreds of tropical origin were found to contain 77% of the total allelic diversity of the collection, whereas the non-stiff stalk and stiff stalk groups were found to present a substantial bottleneck, with only 48% and 42% of the total allelic diversity, respectively, being present. Of the total number of polymorphic SNPs, only about 35% were shared between all three of the groups (Figure 5). Another difference between stiff stalk/non-stiff stalk and the remainder of the collection was a shift in the MAF distribution, with more than half of their SNPs (68% and 59%, respectively) having a MAF greater than 0.1. By contrast, the Goodman association panel captured 75% of the total allelic diversity and was highly representative of the entire collection, with an SFS similar to that obtained using all the samples. The diverse panel formed by the 27 maize inbred founders of NAM and IBM contained 57% of the overall allelic diversity, showing that, even with a very small number of samples, NAM captured more than half the total allelic diversity present in the inbred line collection.

**Figure 6:**
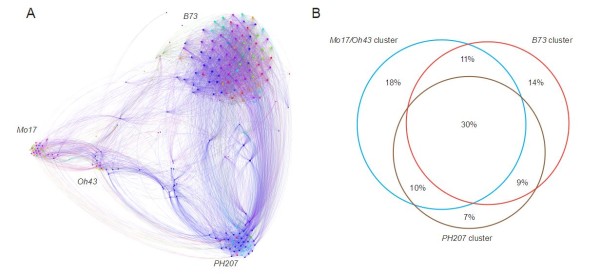
**Expired Plant Variety Protection **(**ExPVP) network diagram and distribution of segregating single-nucleotide polymorphism (SNPs)**. (A) Network of relationships for the ExPVP inbreds constructed using identical by state (IBS) values greater than 0.9. Each dot (inbred line) has a different color assigned based on the company where it was developed. **(B) **Distribution of the segregating SNPs between the three heterotic groups that form the three main clusters in the network graph.

Both Canadian and USA public breeding efforts have successfully incorporated genetic diversity. Collectively, those inbred lines contained 83% of the total allelic diversity of the collection. However, only a modest amount of this diversity has been commercially exploited, and proprietary germplasm with Expired Plant Variety Protection (ExPVP) contains only 45% of the total number of polymorphic SNPs. Moreover, private breeding efforts have favored the divergence between three main heterotic pools (stiff stalk, non-stiff stalk, and iodent). In analyzing the network relationships for the ExPVP inbreds, only 2% of the pairwise IBS relationships with greater than 90% IBS were found to be between inbreds from different heterotic pools (Figure 6A), and only 30% of the total SNPs segregating in the ExPVP materials were shared between all three groups of germplasm (Figure 6B).

We also analyzed pairwise fixation indexes (Fst) between different groups of accessions. The small Fst estimates, averaging only 0.06, indicated that there is moderate differentiation [22] between tropical, stiff stalk, and non-stiff stalk maize populations. Analysis of pairwise Fst and average nucleotide divergence between different USA breeding programs (Table 1) confirmed the picture obtained by analyzing genetic distances. Most of those programs used similar sources of diversity, with an average pairwise Fst of 0.04. Although the maximum values for nucleotide divergence between programs differed, the average values for all the comparisons were around 0.14 (Table 1). The main commercial companies, responsible for most of the maize cultivated in the USA, have had very similar strategies when deciding which sources of germplasm will benefit their breeding programs and, based on the data obtained from their ExPVP, their populations differ genetically by only 3%. They also had the smallest value for average nucleotide divergence (0.13).

**Table 1:** Pairwise differences between maize breeding programs in the USA.^a^

	IL	IN	IA	MI	MN	MO	NE	NC	ND	W	Mon	Pion
Illinois (IL)		0.14 0.96	0.14 0.99	0.14 0.98	0.14 0.97	0.15 0.98	0.14 0.98	0.14 0.98	0.15 0.93	0.15 0.98	0.14 0.98	0.14 0.95
Indiana (IN)	0.01		0.14 0.99	0.14 0.95	0.14 0.98	0.15 0.99	0.14 0.96	0.14 0.96	0.15 0.92	0.15 0.96	0.14 0.96	0.14 0.96
Iowa (IA)	0.01	0.01		0.14 0.93	0.14 0.99	0.15 0.99	0.13 0.99	0.14 1.00	0.15 0.99	0.14 0.98	0.13 0.99	0.14 0.96
Michigan (MI)	0.01	0.02	0.03		0.14 0.97	0.15 0.93	0.14 0.91	0.15 0.91	0.15 0.93	0.15 0.97	0.14 0.95	0.14 0.92
Minnesota (MN)	0.02	0.02	0.02	0.02		0.15 0.96	0.14 0.98	0.15 0.99	0.15 0.94	0.14 0.99	0.14 0.98	0.14 0.95
Missouri (MO)	0.02	0.02	0.03	0.03	0.03		0.14 0.97	0.15 0.95	0.15 0.92	0.15 0.99	0.14 0.99	0.14 0.96
Nebraska (NE)	0.04	0.04	0.03	0.06	0.05	0.05		0.14 0.99	0.15 0.89	0.14 0.97	0.13 0.98	0.13 0.95
North Carolina (NC)	0.05	0.05	0.04	0.06	0.05	0.04	0.06		0.15 0.90	0.15 0.93	0.14 0.98	0.14 0.95
North Dakota (ND)	0.03	0.03	0.04	0.03	0.02	0.03	0.08	0.06		0.15 0.94	0.15 0.94	0.15 0.88
Wisconsin (WI)	0.02	0.03	0.03	0.03	0.02	0.04	0.07	0.07	0.03		0.14 0.98	0.15 0.94
Monsanto (Mon)	0.04	0.03	0.02	0.05	0.03	0.05	0.05	0.04	0.07	0.06		0.13 0.99
Pioneer (Pion)	0.04	0.04	0.03	0.05	0.04	0.04	0.06	0.06	0.07	0.06	0.03	

^a^Lower diagonal shows pairwise Fst estimates between USA breeding programs, whereas upper diagonal shows average nucleotide divergence and maximum nucleotide similarity.

Within chromosomes, all groups consistently displayed smaller values of Fst and lower MAF in the pericentromeric regions versus the remainder of the genome.

### Genetic diversity

**Figure 7:**
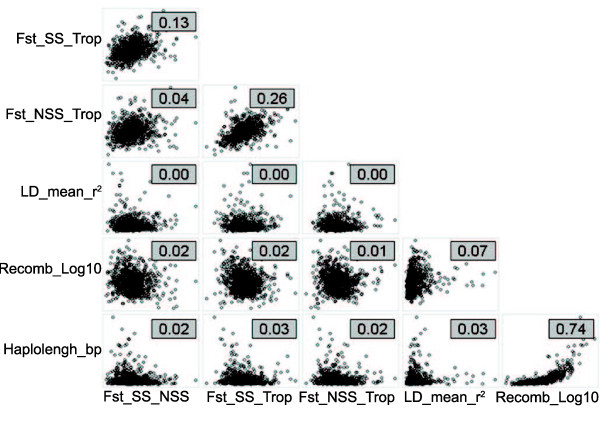
**Genome-wide pairwise relationships between different genetic diversity measurements**. Relationships between nested association mapping (NAM) recombination rate (log_10 _cM/Mb), average haplotype length (bp), average LD (*r*^2^), and fixation indexes (Fst) between stiff stalk, non-stiff stalk, and tropical lines at the NAM genetic map bin scale. The numbers indicate the coefficient of determination (*r*^2^) calculated using Spearman's rank correlation. LD, linkage disequilibrium.

To evaluate the levels of diversity and divergence in the entire collection and within different groups of germplasm, we calculated LD, haplotype length, and population differentiation (Fst) across the entire maize genome. We also calculated the correlation between those measurements and previous recombination rates across the genome estimated with NAM [23] (Figure 7).

**Figure 8:**
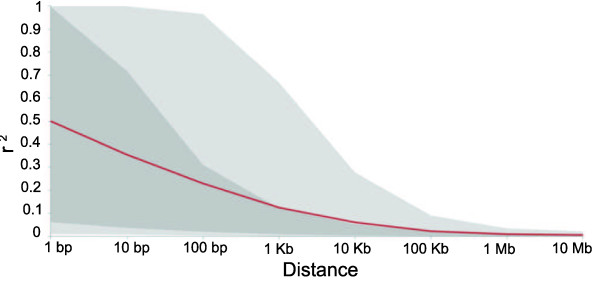
**Decline of genome-wide linkage disequilibrium (LD) across all maize inbreds**. Mean LD decay measured as pairwise *r*^2 ^between all single-nucleotide polymorphisms in the collection. The red line represents the average value while the darker gray area represents the 50% range of values and light gray 90%.

LD decayed very rapidly within the entire collection, and reached an average *r*^2 ^of 0.2 within about 1 Kb (Figure 8), but the variance is large because the level of LD is dependent on the particular group of germplasm and region of the genome, as can be seen with the differences for the median value for *r*^2 ^within diverse groups of germplasm (see Additional file 4). LD decay was slower within the stiff stalk, non-stiff stalk, and ExPVP groups, for which an average *r*^2 ^of 0.2 was not reached until a distance of approximately 10 Kb. Tropical materials displayed the fastest decay of LD with values similar to the overall sample.

The average GBS marker haplotype length, estimated around each SNP as the number of contiguous SNPs that two random lines from a group share, extending from a focal point forward in both directions, was 52 SNPs (around 1.4 Mb) for the entire collection, with a smaller length within the tropical materials (44 SNPs) and a much larger length in the non-stiff stalk (152 SNPs) and stiff stalk (495 SNPs) groups. The ExPVP group also displayed a large average haplotype length of 200 SNPs (around 5.1 Mb), with mean haplotype lengths greater for lines developed by breeding programs now owned by Monsanto than for Pioneer lines. Core collections such as the Goodman association panel or NAM parents, which were selected to maximize diversity, had the smallest haplotype lengths (81 and 48 SNPs, respectively) (Table 2). Haplotype lengths for the overall sample showed high correlation with the estimates of the recombination rates in NAM (Spearman correlation *r*^2 ^=0.74) (see Additional file 5, Figure 7).

**Table 2:** Average haplotype length for different groups of germplasm.^a ^

Type	Chromosome number										Mean
		
	1		3	4	5	6	7	8	9	10	
All maize	49.8	49.7	53.0	58.9	49.8	51.0	52.3	51.6	48.7	57.8	52.3

Tropical	51.9	43.0	43.9	46.5	43.5	38.1	43.0	43.5	42.6	43.3	43.9

Stiff stalk	494.4	493.3	546.6	523.5	432.9	527.8	410.5	488.9	388.4	647.0	495.3

Non-stiff stalk	170.5	135.7	149.0	154.1	164.2	123.6	156.5	132.6	144.9	190.7	152.2

ExPVP^b^	200.8	203.0	170.8	216.1	192.5	186.0	179.4	209.3	168.8	277.4	200.4

Monsanto	268.4	384.9	246.0	327.4	318.0	253.6	221.7	277.1	232.2	333.8	286.3

Pioneer	223.6	139.6	167.8	226.5	170.5	198.9	206.7	175.6	188.3	267.2	196.5

Association panel	79.6	79.8	90.1	87.4	76.3	81.7	75.3	81.9	76.2	86.4	81.5

NAM^c^	45.0	45.4	53.6	57.0	47.9	43.3	43.3	52.3	43.4	52.1	48.3

^a^Number of sites defining a haplotype by chromosome calculated using genotyping by sequencing; markers for different groups of germplasm.

^b ^Expired Plant Variety Protection

^c ^Nested association mapping

None of the other correlations tested was strong, probably because of the large diversity of the sample and large physical size of the NAM genetic map bins (average of 2.4 Mb). However, the fixation indexes between both temperate groups and tropical materials showed an *r*^2 ^of 0.26, indicating common allele frequency differences between groups, probably related to the adaptation bottleneck.

In addition, when analyzing the entire chromosome with all samples, chromosome 4 was found to have a larger haplotype length (sites) compared with the rest of the chromosomes (Table 2). When looking at physical distance (in Mb), this increase was consistent in all groups. One region on chromosome 4 that seemed to increase the average haplotype length is located between 40 and 65 Mb, a region with important genes related to the domestication and improvement processes [24,25]. This region also showed lower diversity and MAF. The stiff stalk, non-stiff stalk, and ExPVP groups also exhibit a longer than average haplotype length for chromosome 10, where one of the major photoperiod response genes is located [26].

### Genome-wide association studies

The germplasm set conserved in the USDA collection is extensive and publicly available, and contains a high amount of allelic diversity and rapid LD decay. For these reasons, we wanted to explore its possible use as a panel to study quantitative traits, combined with a strategy of low-coverage data in multiple samples. We used a simple Mendelian trait, namely, kernel color, with an approximate frequency of 20% for white kernels in our population, to perform GWAS using GBS markers. The SNP with strongest association (*P *= 10^-86^) with kernel color was found within the *Y1 *gene that reduces the presence of carotenoid pigments in the endosperm [27] (see Additional file 6, Figure 9).

**Figure 9:**
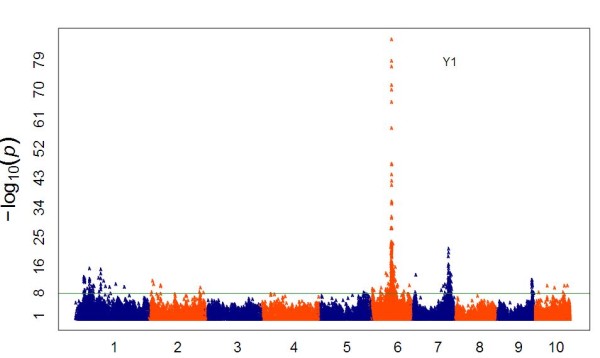
**Genome-wide association study (GWAS) for yellow versus white kernels**. GWAS for kernel color on 1,595 maize inbred lines with yellow or white kernels.

Because the power to detect alleles at lower frequencies is expected to be less, we decided to test another Mendelian trait, sweet corn versus starchy corn, where the sweet phenotype is present at a much lower frequency (5%) than the white kernel type. This trait has been affected by strong selection pressure, both during domestication and the breeding process [28], resulting in an extensive block of elevated LD surrounding the targeted area, especially when the inbred is a dent line that has been converted into a sweet line. The two SNPs with strongest association (*P *values between 10^-61 ^and 10^-52^) defined a 14 Mb interval containing *Su1*, a gene that participates in kernel starch biosynthesis [29] (see Additional file 7, Figure 10).

**Figure 10:**
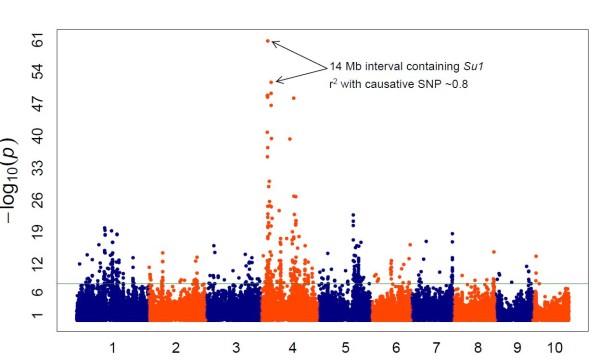
**Genome-wide association study (GWAS) for sweet versus starchy corn**. GWAS for kernel color on 2,145 maize inbred lines with sweet or starchy kernels. SNP, single-nucleotide polymorphism.

Finally, we tested the power of this association panel with a complex trait, the number of growing degree days from planting to the day that 50% of the plants show silk (see Additional file 8, Figure 11). The best association, with *P *= 10^-23^, lies about 2 Kb from *ZmCCT*, an important gene related to photoperiod response and flowering time in maize [26]. The second strongest associations (*P *values between 10^-18 ^and 10^-14^) are located on chromosome 8, surrounding the region where *Vgt1*, one of the major flowering time QTL for maize is located [30]. The next best hit on chromosome 3 (*P *= 10^-14^) does not have any identified candidate gene association, but overlaps with one of the flowering time QTL detected using NAM [31]. A chromosome 7 hit (*P *= 10^-12^) also overlaps with one of the NAM flowering time QTL [31] and is close to the maize flowering time gene *DLF1-DelayedFlowering1 *[32] and the GRMZM2G017016 gene, a putative orthologue of the *Arabidopsis FRI-Frigida *gene [33]. The fifth best hit, on chromosome 1, is located near a very interesting suite of genes spread across a 3 Mb interval, where *teosinte-branched1 *and *dwarf8 *flank one side, while *PhytochromeA1 *flanks the other side [34]. A gene, GRMZM2G144346, containing a *CCT *domain is also located in the region, only 0.2 Mb away from our hit. Recent work has suggested that *dwarf8 *has been a target of selection in early flowering lines [35,36], but it is unlikely to directly contribute much to flowering time [37]. These regions certainly warrant further study.

**Figure 11:**
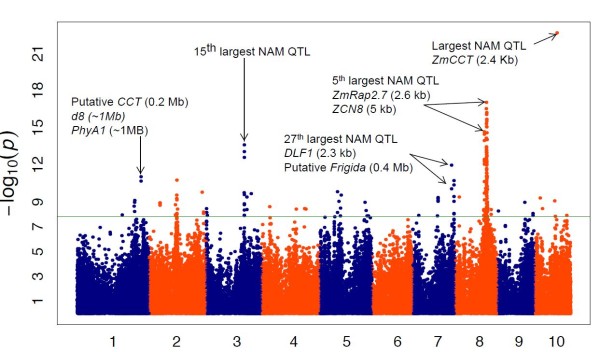
**Genome-wide association study (GWAS) for growing degree days to silking**. GWAS for growing degree days to 50% silking on 2,279 maize inbred lines. NAM, nested association mapping; QTL, quantitative trait loci.

## Discussion

The challenges currently facing agriculture, that is, rapid human population growth, climate change, and the need to balance increasing production with reduced environmental effects, make it necessary to optimize the use of available resources. Genomic data can be used to address these challenges by helping breeders to compare individual plant genomes and optimize the characterization, discovery, and use of functional genetic variation [38]. Germplasm banks around the world curate thousands of maize accessions that, in combination with genomic data, can be explored through GWAS or GS, and could potentially be used for improving agriculturally significant quantitative traits. Inexpensive methods to obtain dense genetic marker information on large samples of germplasm are needed to take full advantage of this tremendous resource [39].

The enormous progress in sequencing technologies that has occurred over the past few years has allowed better understanding of the maize genome. High-density genome sequencing has been used to study maize diversity [4,23-25]. In addition, several studies [39-42] have taken advantage of recently developed SNP genotyping arrays for maize, which have evolved quickly from only a few thousand SNPs to more than 50,000. Although high-density genome sequencing can provide a larger number of markers and a more accurate vision of the genome, its expense has restricted it to only a few hundred samples per study. SNP arrays are cheaper and can analyze larger samples of germplasm; however diversity studies can be confounded by the fact that SNPs are developed using reference sources of diversity, which may cause an important ascertainment bias (Ganal *et al *[19] describes an example with *B73 *and *Mo17 *in the maizeSNP50 chip). GBS has been shown to be a less expensive method for genotyping large numbers of samples, and provides many more SNPs than do SNP arrays. Although the use of a reference genome for calling SNPs from GBS data might cause bias and underestimate the amount of diversity from the groups more distant from the reference, the diversity picture obtained when analyzing the distance matrix seems to be closer to the expectations from simple sequence repeats studies [8], whole-genome sequencing, and maize domestication data [23] than that obtained with SNP arrays.

The percentage of missing data from GBS with enzymes such as *Ape*KI and the levels of coverage obtained here may be a problem for some applications, especially GWAS and GS. Although better coverage can be achieved with more repetitions of the samples, this will increase cost, and quickly reaches a point where there is little reduction in missing data with increased investment in repeated sequencing runs. Given the importance of PAV in maize [2,3,24,43] some of the missing data are very probably due to the absence of some regions of the *B73 *genome in other inbred lines. As shown here, simple imputation procedures based on identifying the most similar haplotype can be used to supply some of those missing data, and this imputation may be sufficiently accurate provided that similar haplotypes are present in the sample of genotypes. This kind of procedure may work better as the total number of maize samples in the GBS database increases, but it may also cause over imputation of data that are actually biologically missing as a result of a PAV. Alternative methods for handling missing SNP data in GBS datasets include an approach that avoids using a reference genome, such as the one recently used for switchgrass [44], or one that genetically maps individual GBS sequence tags as dominant markers [13].

Another important difference between the results obtained with GBS and the results from SNP array methods seems to be the MAF distribution. Whereas array assays seem to oversample SNPs with intermediate frequencies [45] even when analyzing diverse maize collections [9,41], more than half of GBS SNPs within our collection are rare (this is especially true within some of the more diverse germplasm groups). As sequencing technologies improve, the number of rare alleles detected is increasing. In humans, recent studies have found that the majority of variable genomic sites are rare, and exhibit little sharing between diverged populations [46]. The importance of rare alleles is not yet completely clear, and further studies to understand the magnitude of their role causing observable phenotypic variation are underway [38]. There are strong arguments both in favor and against the rare allele model, which hypothesizes that quantitative traits are largely controlled by rare alleles of large effect [15,17].

GWAS studies have shown that variation in some traits is related to rare alleles, and that those rare variants could explain an additional fraction of the missing heritability [15]. However, identifying rare variants through GWAS is challenging, and requires large sample sizes [38]. With the present work, we present an extensive genetic characterization of the maize inbred lines preserved by one of the largest crop germplasm banks in the world, using a method that detects rare alleles with high confidence levels. Moreover, our data show that when there are not enough resources to extensively evaluate the entire collection, a smaller number of samples (such as the maize association panel or even the NAM parents), can, if chosen based on appropriate criteria to maximize haplotype diversity, capture a high portion of the rare alleles, allowing detection of rare allele effects that may be desirable to incorporate into breeding programs.

A complication of using the entire USDA-ARS maize inbred collection for breeding or GWAS is the close relationships between some of the lines. When the seed yield of a few inbreds derived from the Iowa Stiff Stalk Synthetic and their derivatives facilitated the transition to single-cross hybrids, these inbreds became the female parents of choice for many breeding programs [47]. For example *B73*, the main founder of the stiff stalk group, is closely related to more than 50 other inbred lines from different programs in the collection. Several germplasm sources were used to generate the male pool (non-stiff stalk). However, the visualization of the genetic relationships through the MDS shows that even if the non-stiff stalk group forms a larger cluster (revealing a higher amount of diversity), an overlap between the stiff stalk and non-stiff stalk group still exists.

As shown by the MDS plot and Fst values, most of the germplasm from classic breeding programs of the Corn Belt region is closely related. The bottleneck is even narrower when ExPVPs are examined. Using a much smaller sample of SNP markers, Nelson *et al. *[48] reported that most of the ExPVPs released in the past three decades could be clustered into six primary groups represented by six prominent public inbred lines. More recently, Mikel [49] studied the pedigree records of several inbreds registered until 2008, and found that the genetic contribution of the inbred *Mo17 *decreased, whereas that of *Oh43 *increased. Our analysis shows that the ExPVP inbreds tend to cluster into three main groups, with *B73, Mo17/Oh43*, and *PH207 *being the principal connectors within each cluster. Although all of the major private seed companies are represented within each group (consistent with the small value of divergence between companies), Pioneer germplasm is represented more in the iodent group (including *PH207*) and more of its germplasm falls outside the three main clusters (*B73, PH207/Oh43*, and *PH207*). This result is in concordance with the observed smaller average haplotype length of Pioneer germplasm.

Although the recycling of elite lines as breeding parents has markedly reduced the amount of diversity used by maize breeders over the past few decades, breeders have also been aware of the importance of maintaining and introducing diversity into their programs [50]. The determination of breeders to search for new sources of promising, exotic germplasm is reflected in the Ames inbred collection. For instance, the GEM program aims to broaden the germplasm base of corn hybrids grown by farmers in the USA [51]. Combining the efforts of public and private cooperators, this project has introduced tropical alleles into elite USA germplasm. Our molecular characterization of these materials shows that the GEM program has been effective, as most of its inbreds lie somewhere between the ExPVPs and tropical materials on the MDS plot. According to our results, other public programs that have succeeded in incorporating tropical diversity into their materials are North Carolina State University and the University of Missouri. On the other side of the graph, adaptation to colder climates has been accomplished using different heterotic pools within the Northern USA and Canadian programs. Overall, although inbred lines from breeding programs from other parts of the globe might have different haplotype combinations (related to the use of different breeding pools), the USA and Canadian public inbred lines preserved at NCRPIS capture most of the total allelic diversity uncovered in this study.

GBS has yielded the greatest number of SNPs ever obtained from a large maize association panel to date. As seen with our GWAS analysis, the data can provide accurate mapping of simple and complex traits for the most important genes. Van Inghelandt *et al. *[52] suggested that with an association panel of 1,537 elite maize inbred lines, 65,000 SNPs should be sufficient to detect associations with the genes with biggest effects. Lu *et al. *[41] used a panel containing tropical and temperate materials, and suggested that 230,000 to 460,000 markers would be needed. However when comparing the results for the two locations with the best flowering time associations in our study, we observed that the most important flowering time gene, *ZmCCT*, was targeted with only one SNP, meaning that it could easily have been missed. By contrast, the *Vgt1 *peak showed more than 80 SNPs associated with the trait (Figure 11). The main difference between these two important QTL is that the *ZmCCT *polymorphism is very rare in temperate materials with very low levels of LD, whereas the *Vgt1 *variation is common in temperate inbred lines that have higher LD. When GBS data are used to perform GWAS, the probability of finding the causative SNPs in the dataset is highly dependent on the trait itself and the germplasm in which it is expressed. The length and number of the haplotypes detected vary enormously, depending on the region of the genome and the germplasm group. Some germplasm groups are currently under-represented in our maize dataset. As a result, population bottlenecks can cause a polymorphism that is not present at an appreciable frequency to pass the GBS pipeline quality filters. Therefore, it is unlikely that a causative polymorphism is present in the GBS dataset if it is unique to one of these germplasm groups. In addition, if the region has high haplotype diversity, rapid LD decay indicates that it is very likely that, even with approximately 700,000 SNPs we might not find a marker in LD with a particular causative polymorphism of interest. This situation is reflected in a large portion of chromosome 10 where the *ZmCCT *gene is located, and tropical inbreds have much greater haplotype diversity than the rest of the collection. This means that, although 700,000 SNP markers are likely to be sufficient for analysis of temperate alleles, they are not sufficient to perform accurate GWAS with tropical alleles.

However, numerous inbreds in the collection are IBD for specific regions, allowing a strategy of accurate imputation. Based on common local haplotypes defined with GBS SNPs, high-density markers for a representative inbred obtained through whole-genome sequencing can be imputed between GBS markers, thereby increasing marker density.

In summary, our GWAS results for days to silking showed that this association panel combined with the GBS information can help to dissect the genetic architecture of important agronomic complex traits. Our best association signals corresponded to regions in which *a priori *candidate genes or previously identified flowering time QTL are located. Nevertheless, identifying the causal gene is complex. Excluding the *ZmCCT *gene hit on chromosome 10, all other major associations contain several SNPs. These hits cover regions that can extend for more than 10 Mb, even though our average LD decays very rapidly. For *Arabidopsis *[53] and rice [54], the results suggest that the occurrence of these 'mountain landscapes' could be related to the presence of several linked genes across the region. In maize, the dissection of a candidate region contributing to flowering time variation on chromosome 6 suggests that a cluster of tightly linked genes are responsible for the phenotypic variation [55]. In our study, the linked associations on chromosome 8 correspond with the position of two known flowering time genes, *ZmRap2.7 *[30] and *ZCN8 *[56]. A similar situation occurs for the hits on chromosome 7 with candidates *DLF1 *and *FRI*. Lastly, on our chromosome 1 region, extended haplotype lengths for some subpopulations and a strong correlation between the region and population structure have been reported [37]. Within 3 Mb, there are genes that have been under selection since the domestication of maize including *tb1 *and *d8 *[25,36] and two strong candidate genes for flowering time (*CCT *and *PhyA1*). All these results for our candidate regions support the hypothesis of the presence of some multigene complexes that may have evolved together during the process of maize domestication and adaptation. Further studies to unravel these regions and better understand the genetic architecture of flowering time are needed. Flowering time and adaptation to temperate climates are complex traits that seem to be controlled by several genes with small effects, organized in clusters across the genome.

## Conclusions

As previous studies have suggested [7,8,39], the genetic diversity preserved at germplasm banks can be a useful resource for breeders and geneticists. Development of new germplasm will benefit from the knowledge of alleles from diverse materials associated with targeted traits [57], and from the methods and tools used to mine and translate this knowledge into products. However, collections may remain a hidden treasure if the amount and distribution of genetic diversity preserved is not understood, preventing users from making the right choices with the available material. With this study, we have provided the maize research community with a new tool that can be used to better understand and manipulate the genetic architecture of complex traits. It will permit more efficient and targeted use of the breeders' work and of the vast amount of diversity available in the USDA-ARS maize germplasm bank. Experimental designs based on particular haplotypes or maximizing the diversity for a determined number of entries may be possible, optimizing the resources available to each researcher.

## Materials and methods

### Sample collection and genetic characterization

Leaf samples from the entire available collection of maize inbred lines conserved at the USDA Plant Introductory extension in Ames (IA), including several sources for the same accession, and from other collaborators, were collected from an experiment planted near Columbia-Missouri (MO) in 2010. Several checks across the experimental design were planted in order to collect accurate phenotypic data. Leaf samples from those checks were also collected to serve as controls during the DNA manipulation process. DNA extractions were performed on leaf punches from a single plant using a commercial kit (DNeasy 96 Plant Kit, Qiagen Inc., Valencia, CA, USA). DNA from the Goodman association panel was provided by the Institute for Genomic Diversity (Cornell University, Ithaca, NY, USA) This panel was sequenced twice to serve as technical replicates for quality control. Another 95 additional samples from the entire collection were selected to maximize diversity, and sequenced several times with the same purpose and as sources of data for imputation.

Genotype data was generated following the GBS protocol [13], using *Ape*KI as restriction enzyme and multiplexing 96 samples on each Illumina flow cell lane. Raw reads from the machine for the samples reported here were analyzed in conjunction with approximately 18,000 additional maize samples, including NAM and other linkage populations. The GBS sequencing data has been submitted to NCBI SRA (study accession number SRP021921). The GBS discovery pipeline for species with a reference genome, available in TASSEL (version 3.0) [58], was used. The pipeline parameters used to filter the SNPs were a minimum SNP call rate of 10%, minimum inbreeding coefficient (coefficient of panmixia, 1-H_O_/H_E_, where H_O _= observed heterozygosity and H_E _= expected heterozygosity) of 0.8, and MAF of 0.2%. For the 'biparental error correction' step that uses the information of biparental populations present in the overall sample, we used a maximum error rate (apparent MAF in biparental families where the SNP is not actually segregating) of 0.01, and a minimum median *r*^2 ^for LD with markers in the local genome region across biparental families of 0.5. For the latter parameter, the *r*^2 ^for each individual biparental family in which a SNP was segregating (minimum MAF of 0.15) was calculated as the median *r*^2 ^in a window centered on the SNP in question and consisting of one-twentieth of the SNPs on the corresponding chromosome. SNPs within 100 Kb of the SNP in question were excluded from the calculation, as they could alter the result because of possible errors in the order of the sequenced bacterial artificial chromosomes.

The imputed data used for the GWAS was generated using a custom Java script that divided the entire SNP dataset into 1,024 SNP windows and looked for the most similar inbred line within each window to fill the missing data. The algorithm takes advantage of small IBD regions shared between pairs of inbred lines in the collection; if the window from the closest neighbor has more than 5% difference from the line being imputed, the data point is left as missing. The entire GBS *Zea *database (approximately 22,000 samples) was used to search for the closest sample.

Both GBS SNP datasets (raw and imputed) are publicly available through Panzea [59]

### Population structure and pedigree relationships

IBS and IBD were calculated for all possible pairwise comparisons using PLINK (version 1.07) [60]. For each individual, the values for the nearest neighbors, based on how similar (IBS) they were, were summarized using the '--cluster --neighbour' option in PLINK. To maintain the assumption of independence between markers for the IBD calculations, SNPs were pruned with a window of 100 adjacent SNPs and a step size of 25 SNPs. The *r*^2 ^threshold was 0.2. The resulting number of remaining SNPs was approximately 200,000.

Network diagrams were generated using the open-source network visualization platform Gephi (version 0.8) [61].

MDS through principal coordinates analysis for two dimensions was performed on the IBS matrix using the isoMDS option of the package MASS from R [62]. Accessions were assigned to a specific group or breeding program according to the information available in the Germplasm Resources Information Network (GRIN) database.

### Distribution of alleles and allele frequencies

MAF were calculated using the 'Geno Summary by Site' analysis tool in TASSEL (version 4.0) [58]. Taxa and site filter tools from that program were also used. To remove possible sequencing errors, only alleles detected in at least two individuals in a particular group were considered to be present for the allelic diversity calculations.

### Genetic diversity

To analyze genetic diversity, each inbred was considered a random sample of a single maize haplotype from the populations being examined. Hence, heterozygous SNP genotypes were set to 'missing'. With the resulting dataset, pairwise IBS for all pairs of individuals from each set of populations being compared was calculated for each 1 Mb window. Average nucleotide difference was defined as 1 minus average IBS. To estimate average haplotype length, we followed the procedure proposed by Hufford *et al.*[25]. Choosing one random starting data point across the genome and two random inbred lines, we compared the genotypes of the two lines at the focal point, extending outward in both directions until we found different genotypes, then we sorted the results according to the median site to calculate the average distribution per interval. Filtering for allele frequency was not applied before this calculation. Consequently, in order to allow for possible sequencing errors, a one-SNP mismatch was permitted on each side of the initial counting site before assigning the end of the haplotype. Pairwise Fst between each group of maize lines were calculated for all the SNPs as described by Weir and Cockerham [63], and an average Fst by Mb window was presented. All genetic diversity calculations were performed using custom Java and R scripts.

For the LD analysis, SNPs with more than 25% missing data and with a MAF less than 0.05 were filtered before the analysis, resulting in a total set of 21,806 SNPs. To avoid the bias that differences in sample sizes of the different populations could cause, one random set of 180 inbreds from each of the tropical, ExPVP, and overall populations was selected. LD was calculated using TASSEL [58], and output report tables from that program were summarized using R.

### Genome-wide association analysis

The GRIN database contains public information for different descriptors for each of its entries. When these analyses were performed, kernel color phenotypes were available for 1,595 accessions (1,281 yellow versus 314 white). We first performed a GWAS for kernel color, with white kernels coded as 0 and yellow as 1. In addition, information about kernel type was used to analyze starchy corn (0) versus sweet corn (1), with 2,520 entries in the first category and 140 into the second. Data on flowering time were collected from plants grown in randomized augmented designs in three environments (Ames, IA; Clayton, NC; and Aurora, NY) during summer 2010. Growing degree days were calculated using climate data from weather stations located near the farms. Best linear unbiased predictors for each line across environments were constructed with ASREML software (version 3.0) [64]. Blocking factors included environment, field nested in environment, and block nested in field. Each field environment error was assumed to be independent and heterogeneous in variance. A first-order autoregressive error term for range and row error structures in each field were also included.

GWAS analyses were performed on the imputed dataset using the GAPIT package for R [65]. For the 10% unimputed (missing) genotypes, the GWAS model assigned an intermediate value before the analysis. For all traits, we used a compressed mixed model [66], where the kinship was calculated as described by VanRaden [67], with a random subset of 10% of the SNPs. The first five principal components calculated with those same SNPs were included as covariates.

## List of abbreviations

BWA: Burrows-Wheeler Alignment; ExPVP: Expired Plant Variety Protection; Fst: Fixation index; GBS: Genotyping by sequencing; GEM: Germplasm Enhancement of Maize; GRIN: Germplasm Resources Information Network; GS: Genomic selection;; GWAS: Genome-wide association study; IBD: Identity by descent; IBS: Identical by state; LD: Linkage disequilibrium; MAF: Minor allele frequencies; MDS: Multidimensional scaling; NAM: Nested association mapping; NCRPIS: North Central Regional Plant Introduction Station; PAV: Presence/absence variation; PCoA: Principal coordinate analysis; QQ: Quantile-quantile; SFS: Site frequency spectrum; SNP: Single-nucleotide polymorphism; USDA-ARS: USA Department of Agriculture - Agricultural Research Service

## Competing interests

The authors declare that they have no competing interests.

## Authors' contributions

MCR analyzed the data and drafted the manuscript. MJM curates the germplasm collection: participated in the design and coordination of the study, and provided phenotypic information. JCG contributed with new analysis tools and analyzed data. JAP analyzed data. KLS and TMC contributed with new analysis tools. RJE, CBA, and SEM coordinated and performed the GBS lab protocol. SAF-G and MDM conceived of the study, participated in the study design and coordination, and collected data. JBH conceived of the study, participated in the project design and coordination, collected data, and helped to draft the manuscript. ESB and CAG conceived of the study, managed its design and coordination, collected data, and helped to draft the manuscript. All authors read and approved the final manuscript.

## Supplementary Material

Additional file 1 **Table S1**. Details for the 2,815 accessions (accession number, number of samples, number of plants, average identical by state (IBS) value for all the samples, percentage of missing data, breeding program, and pedigree group). Click here for file

Additional file 2 **Table S2**. The 10 closest neighbors for each unique entry in our maize list based on identical by state (IBS) values. IBS value for each neighbor is presented between brackets. Click here for file

Additional file 3 **Figure S1**. Network diagram showing the relationships of maize inbred lines with identical by state (IBS) values greater than 0.96. Click here for file

Additional file 4 **Figure S2**. Median linkage disequilibrium (LD) decay measured as pairwise *r*^2 ^between all single-nucleotide polymorphisms (SNPs) in the collection. Each line represents a different group of germplasm. Click here for file

Additional file 5 **Figure S3**. Relationships between nested association mapping (NAM) recombination rate (log_10 _cM/Mb), average haplotype length (bp), average linkage disequilibrium (LD) (*r*^2^), and fixation index (Fst) between stiff stalk, non-stiff stalk, and tropical lines at the NAM genetic map bin scale for each chromosome. The numbers indicate the coefficient of determination (*r*^2^) calculated using Spearman's rank correlation. Click here for file

Additional file 6 **Figure S4 **Quantile-quantile (QQ) plot for kernel color genome-wide association study (GWAS) analysis. Click here for file

Additional file 7 **Figure S5 **Quantile-quantile (QQ) plot for sweet corn genome-wide association study (GWAS) analysis. Click here for file

Additional file 8 **Figure S6 **Quantile-quantile (QQ) plot for flowering-time genome-wide association study (GWAS) analysis. Click here for file
