# Supplementary figures and images for: Comprehensive genotyping of the USA national maize inbred seed bank
Source: Genome Biol. 2013 Jun 11;14(6):R55. doi: 10.1186/gb-2013-14-6-r55 (PMC3707059; doi:10.1186/gb-2013-14-6-r55)

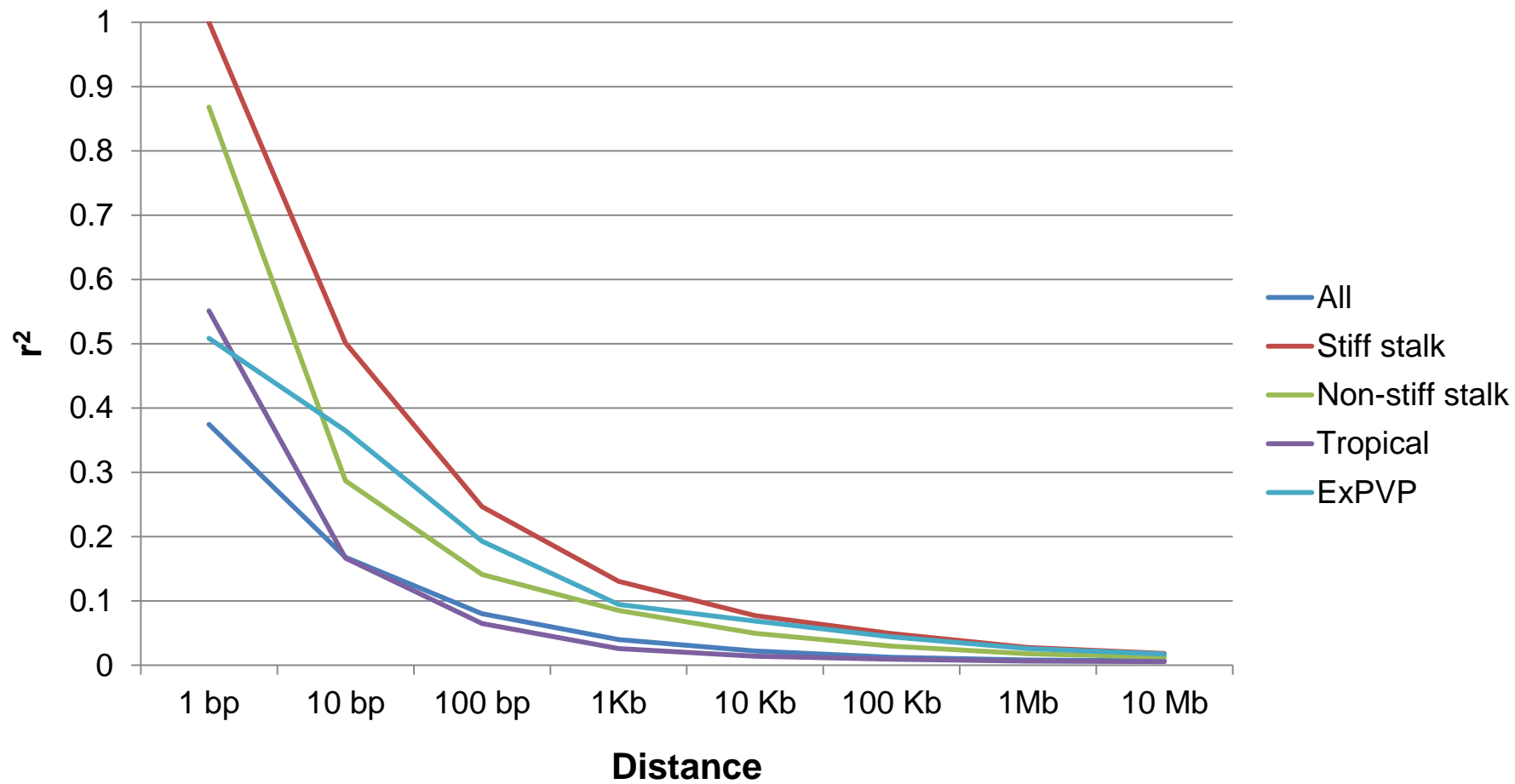

Supplement: Additional file 4 — Figure S2. Median linkage disequilibrium (LD) decay measured as pairwise r2 between all single-nucleotide polymorphisms (SNPs) in the collection. Each line represents a different group of germplasm. [file gb-2013-14-6-r55-S4.PDF]

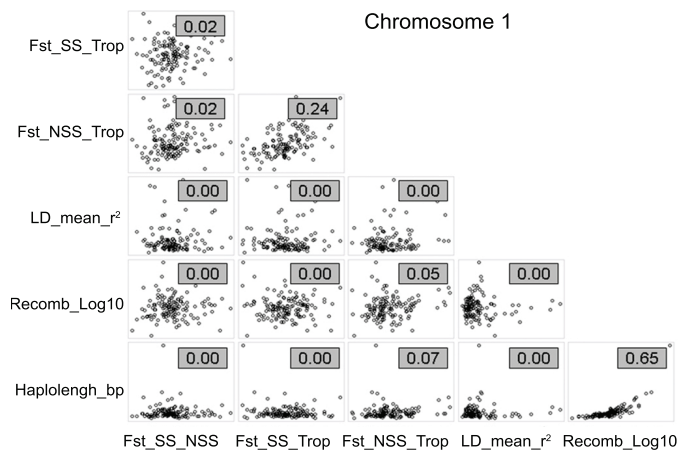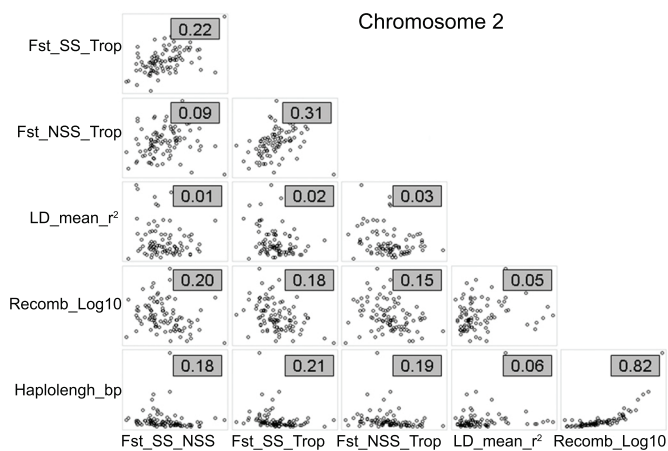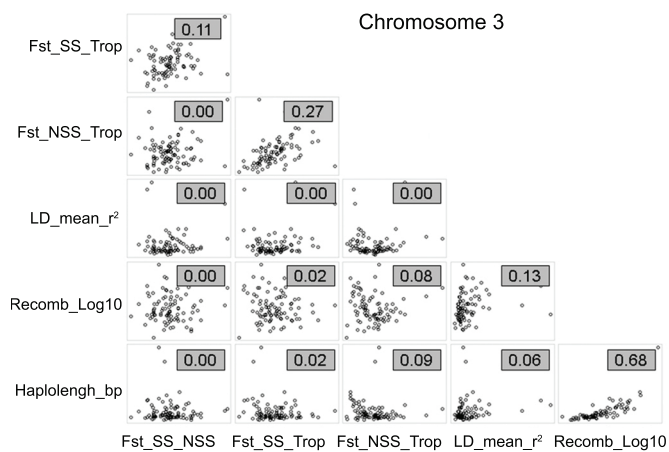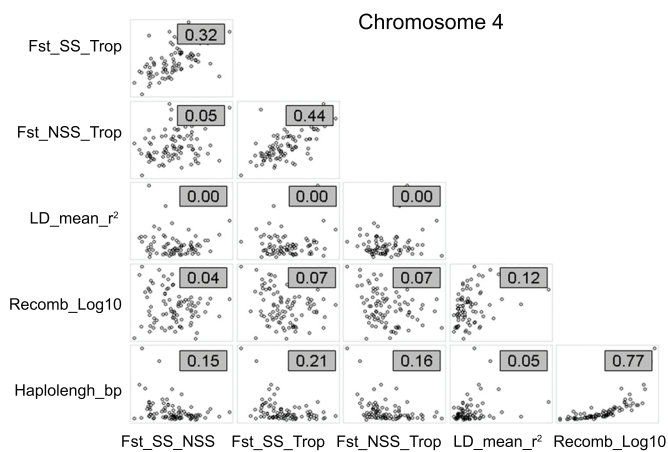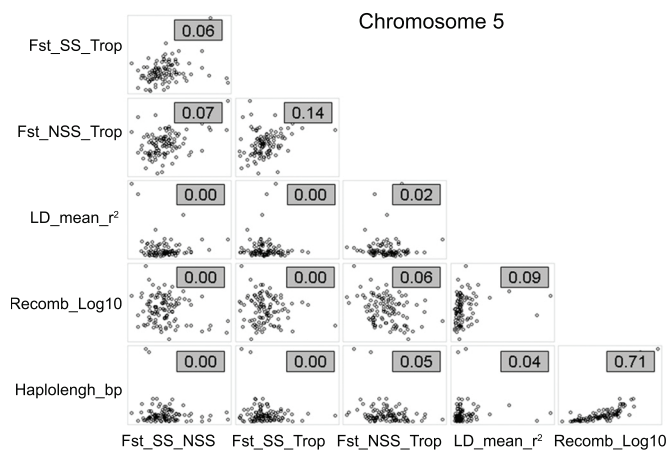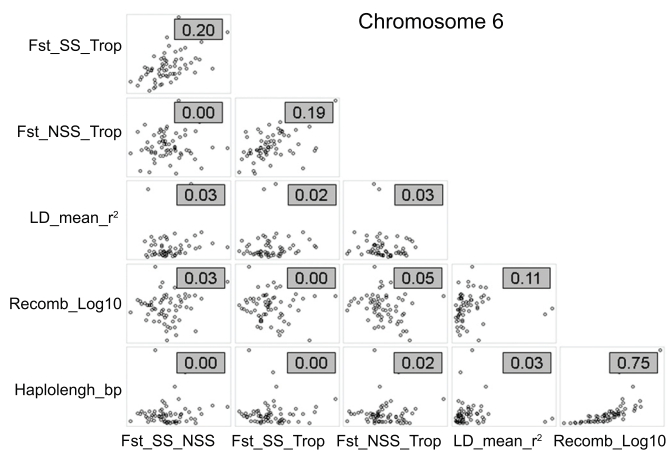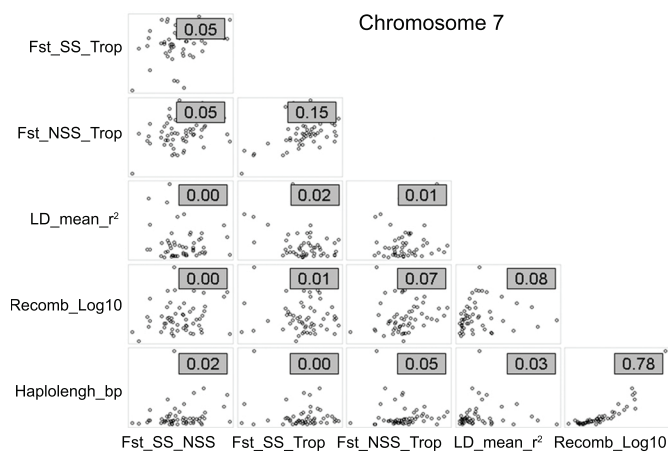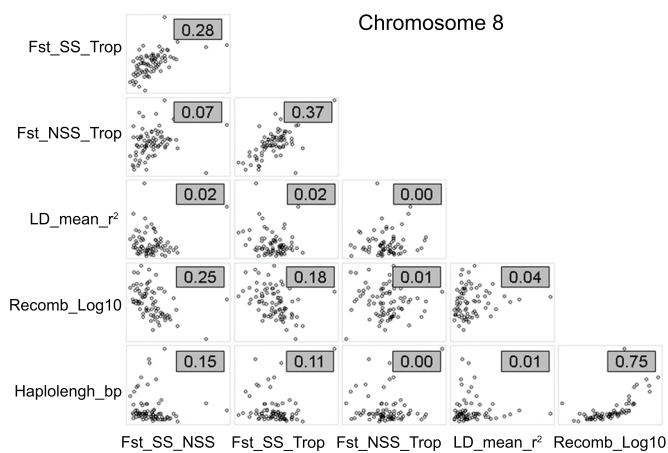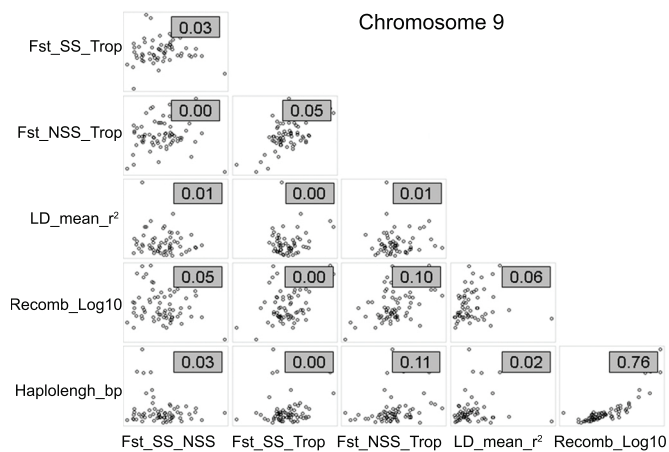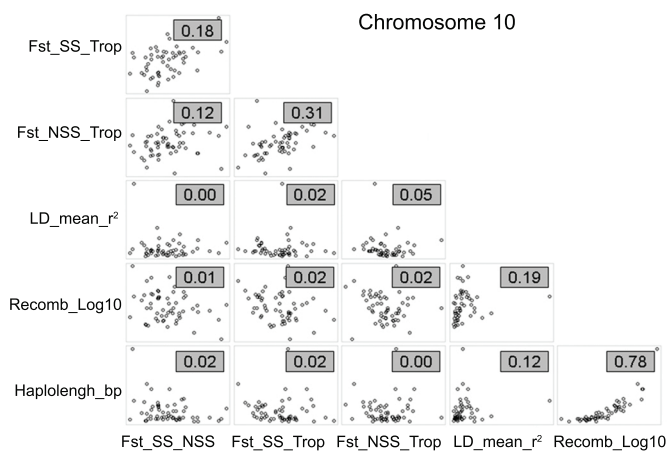

Supplement: Additional file 5 — Figure S3. Relationships between nested association mapping (NAM) recombination rate (log10 cM/Mb), average haplotype length (bp), average linkage disequilibrium (LD) (r2), and fixation index (Fst) between stiff stalk, non-stiff stalk, and tropical lines at the NAM genetic map bin scale for each chromosome. The numbers indicate the coefficient of determination (r2) calculated using Spearman's rank correlation. [file gb-2013-14-6-r55-S5.PDF]

Kernel\_color

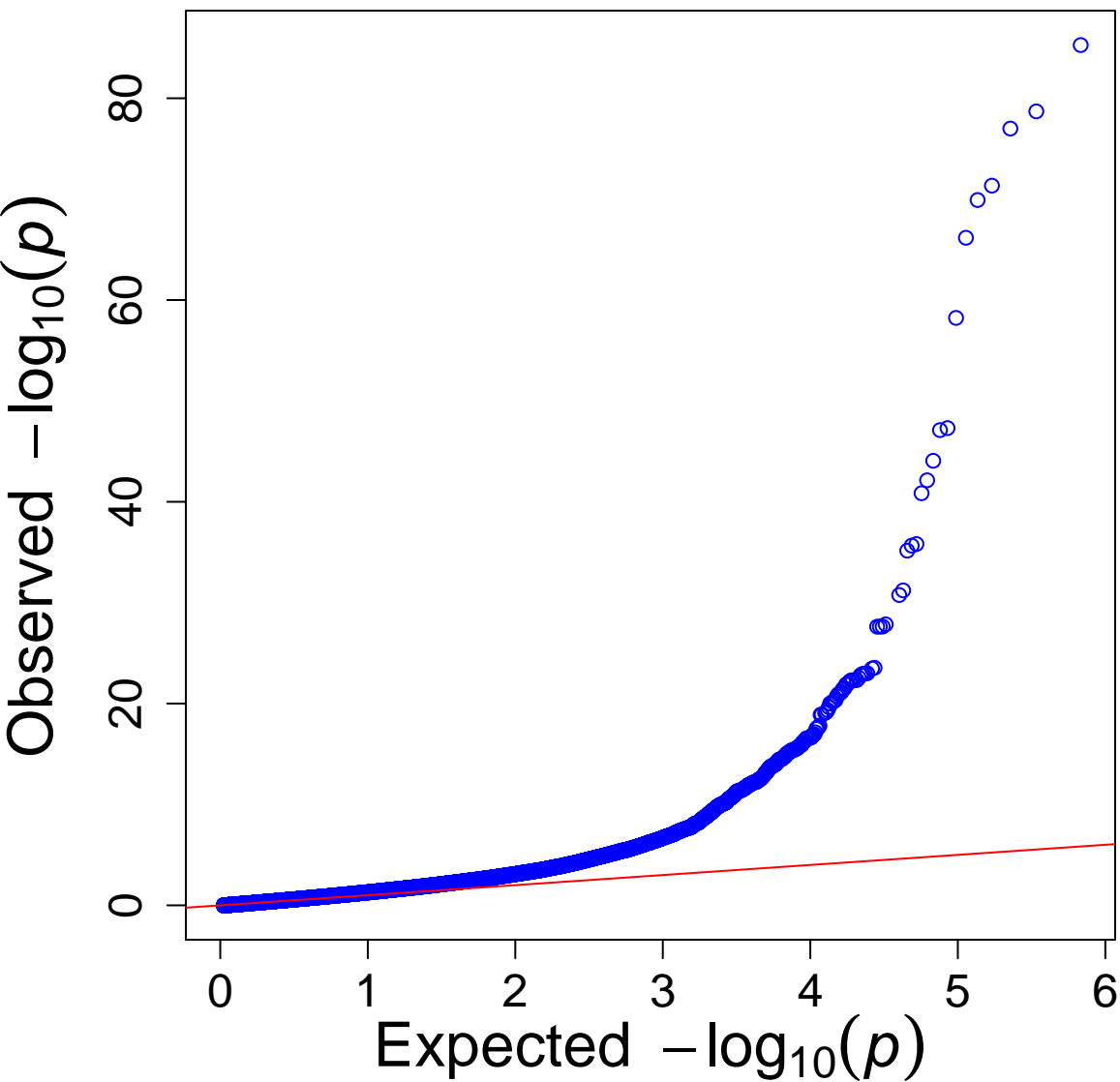

Supplement: Additional file 6 — Figure S4 Quantile-quantile (QQ) plot for kernel color genome-wide association study (GWAS) analysis. [file gb-2013-14-6-r55-S6.PDF]

Sweet

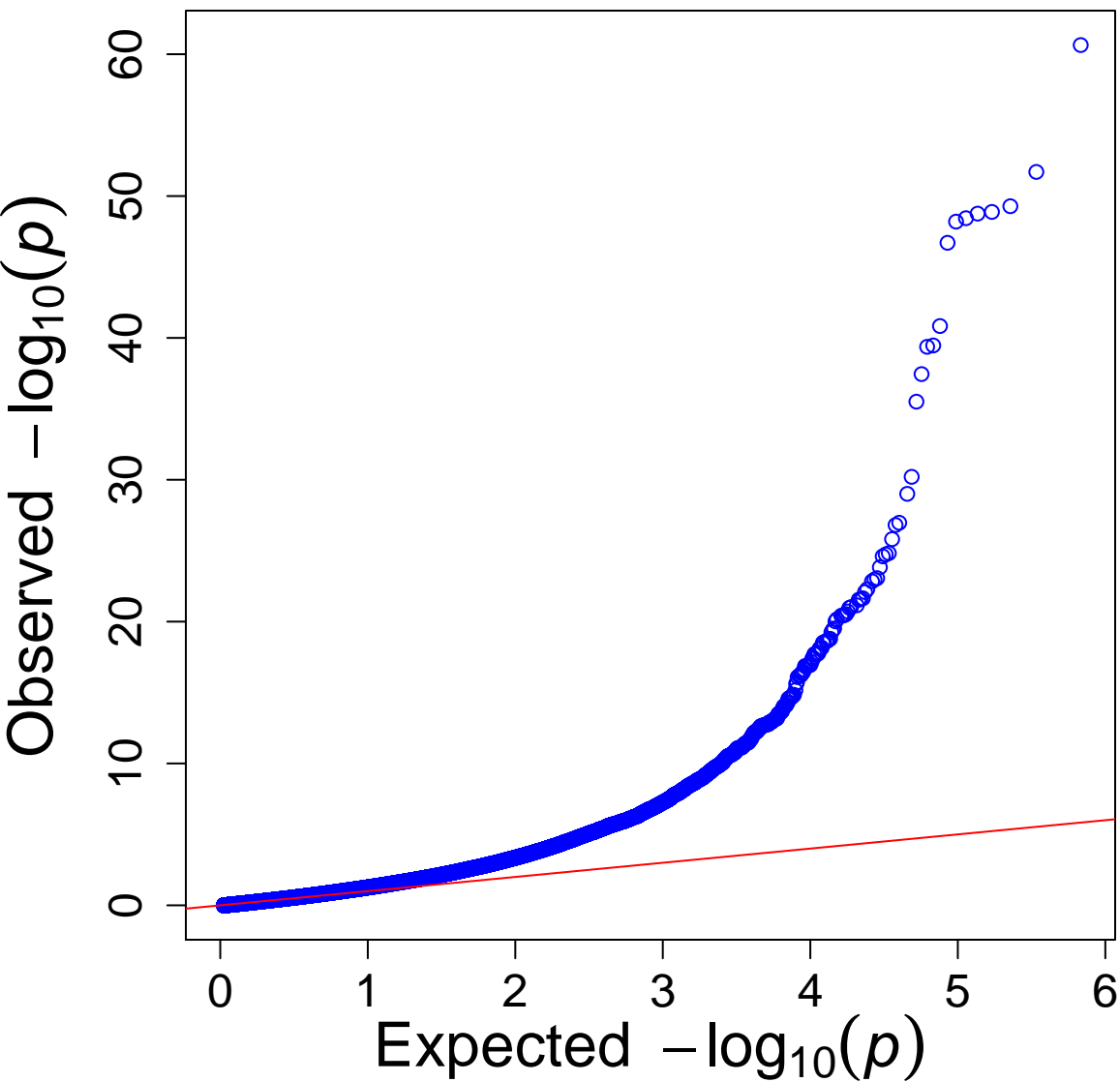

Supplement: Additional file 7 — Figure S5 Quantile-quantile (QQ) plot for sweet corn genome-wide association study (GWAS) analysis. [file gb-2013-14-6-r55-S7.PDF]

# GDD\_DTS

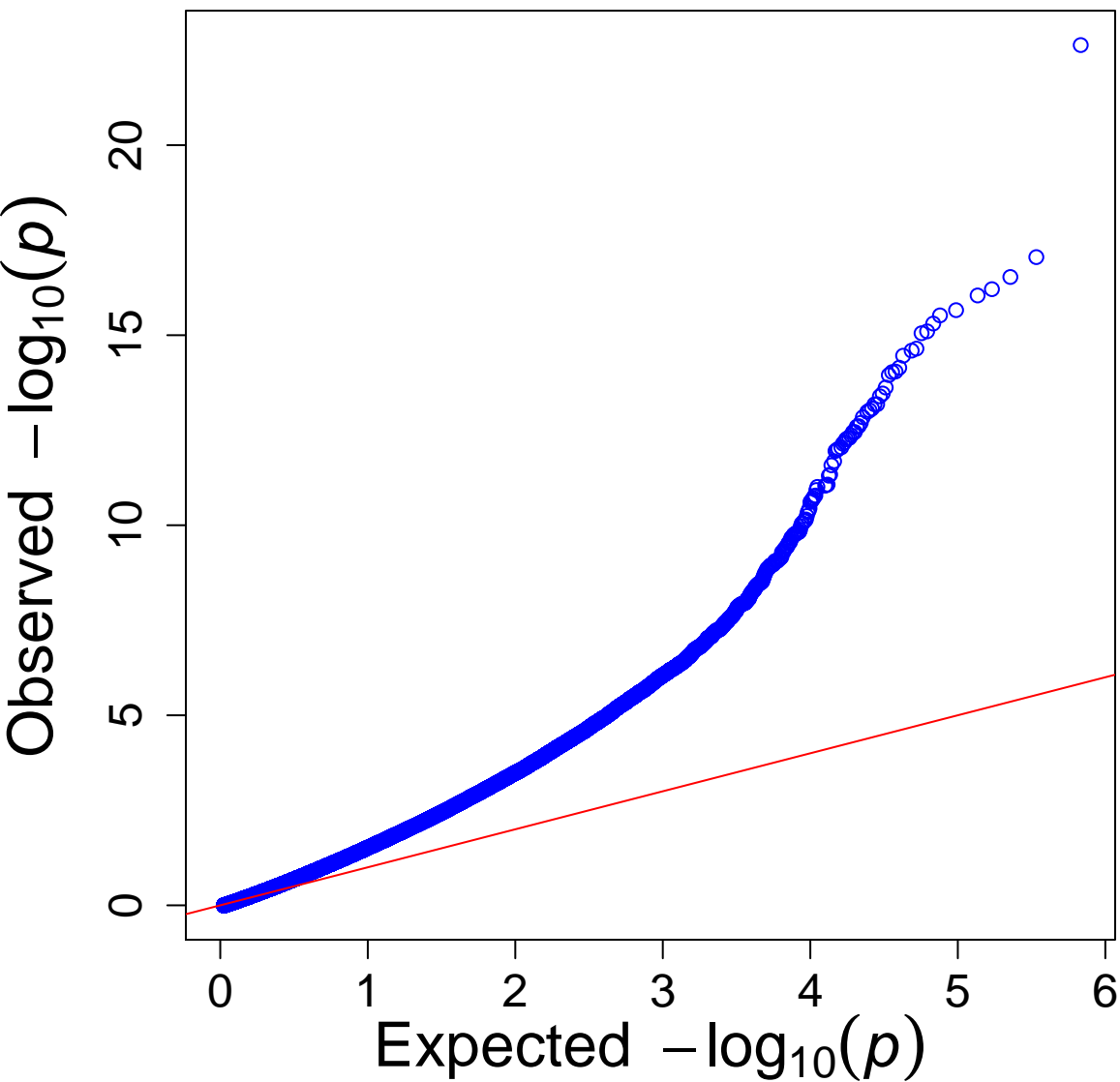

Supplement: Additional file 8 — Figure S6 Quantile-quantile (QQ) plot for flowering-time genome-wide association study (GWAS) analysis. [file gb-2013-14-6-r55-S8.PDF]
