# Supplementary material for: Comprehensive genotyping of the USA national maize inbred seed bank
Source: Genome Biol. 2013 Jun 11;14(6):R55. doi: 10.1186/gb-2013-14-6-r55 (PMC3707059; doi:10.1186/gb-2013-14-6-r55)

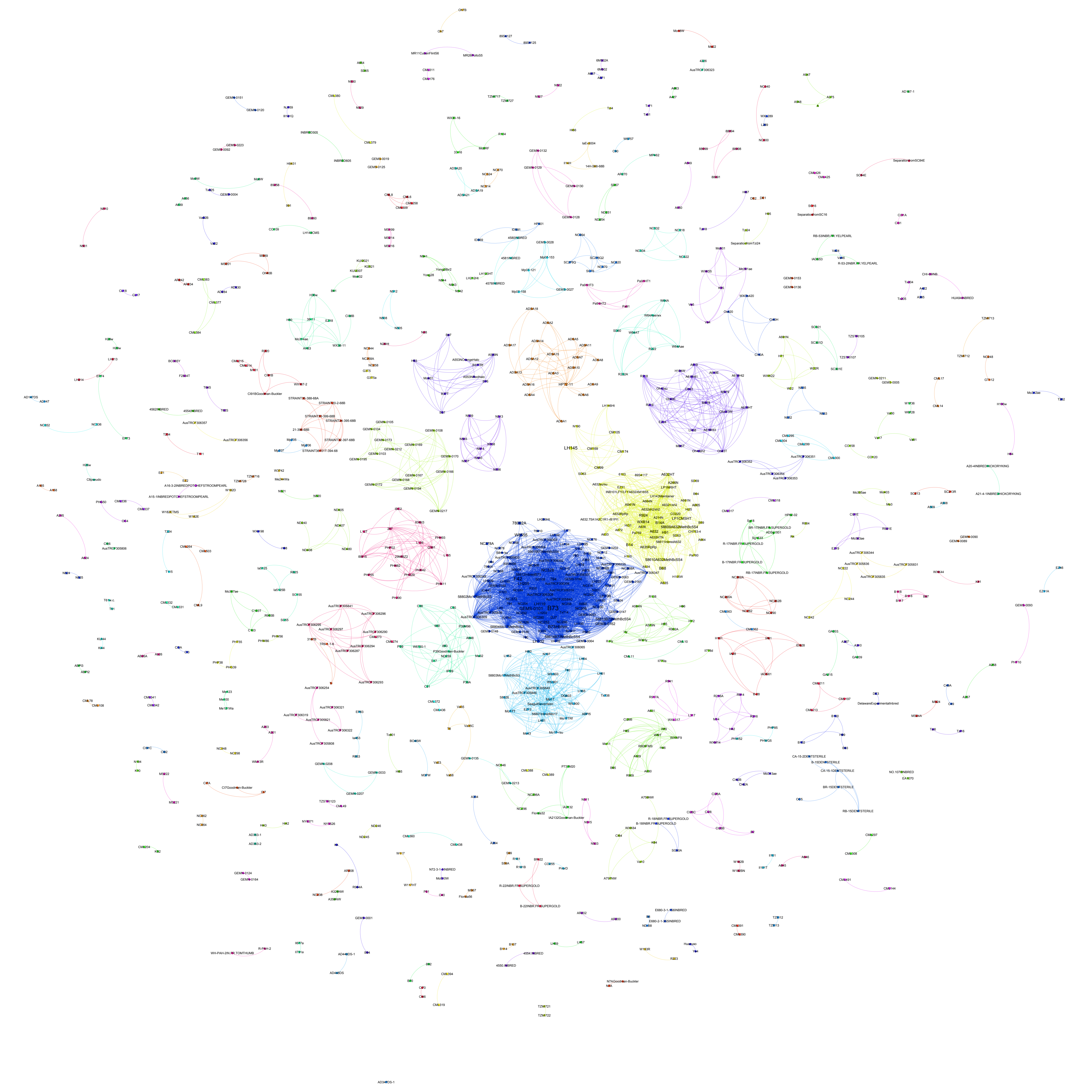

Supplement: Additional file 3 — Figure S1. Network diagram showing the relationships of maize inbred lines with identical by state (IBS) values greater than 0.96. [file gb-2013-14-6-r55-S3.PDF]
